# Is it worthwhile to measure bone quality in patients with adolescent idiopathic scoliosis?

**DOI:** 10.1186/1748-7161-9-S1-O14

**Published:** 2014-12-04

**Authors:** Xuan Zhou, Qing Du, Jianan Li, Xiaohua He, Li Zhao, Peijie Chen

**Affiliations:** 1 Department of Rehabilitation of Xin Hua Hospital Affiliated to Shanghai Jiao Tong University School of Medicine, Shanghai, China; 2 Rehabilitation Department of Jiangsu Province Hospital, Jiangsu Province, China; 3 Palmer College of Chiropractic, Florida, USA; 4 Department of Pediatric Orthopedics of Xin Hua Hospital Affiliated to Shanghai Jiao Tong University School of Medicine, Shanghai, China; 5 Department of Kinesiology of Shanghai University of Sport, Shanghai, China

**Keywords:** Idiopathic scoliosis, Bone strength, Quantitative ultrasound, Female adolescents, Menstruation, Risser’s sign.

## Background

The application of quantitative ultrasound measurement in the studies of AIS is still sparse.

## Aim

The aims of this study were to compare bone quality (speed-of-sound, SOS and z-scores) between female adolescent idiopathic scoliosis (AIS) patients and controls using quantitative ultrasound examination, and further to analyze the relationship between bone strength and maturation, severity and type of scoliosis in AIS patients measured by the same technique along the long axis of the distal radius.

## Design

Case series.

## Methods

88 female AIS patients and 58 healthy female controls from 10 to 16 years of age were included. Quantitative ultrasound measurements were performed at the left distal end of the radius, and the standard method to estimate speed of sound was recorded. Z-score was then calculated. Comparisons were made between the values of SOS and z-score values in patients and age-matched controls.

## Results

The SOS values of 88 female AIS patients were significantly lower than age-matched adolescent controls (P<0.01). However, there was no statistical correlations between bone density and types of scoliosis, as well as family history (p>0.05). The SOS values among different severity groups of curvature were found to be significant, particularly between 10 to 20 degrees and 20 to 40 degree groups, but there was no significant correlation between SOS and Cobb angles. Statistically significant correlations were also found between pre- and post-menarche status. There was significant difference in the SOS values in different Risser stages (p <0.05), and more skeletally immature patients were more osteopenic.

## Conclusions

Comparing to non-scoliotic controls, female AIS patients have generally lower bone quality measuring by quantitative ultrasound. Slower maturation may be one of the factors that affect the bone quality in these patients. Different types of scoliosis and family history have no effect on the bone quality in these patients. Although there were significant differences between SOS values and Cobb angles, this may be due to slower bone maturation than the severity of the AIS curve. It is recommended that quantitative ultrasound measurement should be undertaken in AIS patients.

